# Reduced immunomodulatory metabolite concentrations in peri-transplant fecal samples from heart allograft recipients

**DOI:** 10.3389/frtra.2023.1182534

**Published:** 2023-07-17

**Authors:** Mark Dela Cruz, Huaiying Lin, Jiho Han, Emerald Adler, Jaye Boissiere, Maryam Khalid, Ashley Sidebottom, Anitha Sundararajan, Christopher Lehmann, Angelica Moran, Matthew Odenwald, Matthew Stutz, Gene Kim, Sean Pinney, Valluvan Jeevanandam, Maria-Luisa Alegre, Eric Pamer, Ann B. Nguyen

**Affiliations:** ^1^ Department of Medicine, Section of Cardiology, University of Chicago Medicine, Chicago, IL, United States; ^2^ Duchossois Family Institute, University of Chicago, Chicago, IL, United States; ^3^ Department of Medicine, Section of Infectious Diseases, University of Chicago Medicine, Chicago, IL, United States; ^4^ Department of Pathology, University of Chicago Medicine, Chicago, IL, United States; ^5^ Department of Medicine, Section of Gastroenterology, University of Chicago Medicine, Chicago, IL, United States; ^6^ Department of Medicine, Section of Pulmonary and Critical Care, University of Chicago Medicine, Chicago, IL, United States; ^7^ Department of Surgery, Section of Cardiac Surgery, University of Chicago Medicine, Chicago, IL, United States; ^8^ Department of Medicine, Section of Rheumatology, University of Chicago, Chicago, IL, United States

**Keywords:** gut microbiome, heart transplant, gut microbial metabolites, short chain fatty acids, secondary bile acid, gut microbial diversity

## Abstract

**Background:**

Emerging evidence is revealing the impact of the gut microbiome on hematopoietic and solid organ transplantation. Prior studies postulate that this influence is mediated by bioactive metabolites produced by gut-dwelling commensal bacteria. However, gut microbial metabolite production has not previously been measured among heart transplant (HT) recipients.

**Methods:**

In order to investigate the potential influence of the gut microbiome and its metabolites on HT, we analyzed the composition and metabolite production of the fecal microbiome among 48 HT recipients at the time of HT.

**Results:**

Compared to 20 healthy donors, HT recipients have significantly reduced alpha, i.e. within-sample, microbiota diversity, with significantly lower abundances of key anaerobic commensal bacteria and higher abundances of potentially pathogenic taxa that have been correlated with adverse outcomes in other forms of transplantation. HT recipients have a wide range of microbiota-derived fecal metabolite concentrations, with significantly reduced levels of immune modulatory metabolites such as short chain fatty acids and secondary bile acids compared to healthy donors. These differences were likely due to disease severity and prior antibiotic exposures but were not explained by other demographic or clinical factors.

**Conclusions:**

Key potentially immune modulatory gut microbial metabolites are quantifiable and significantly reduced among HT recipients compared to healthy donors. Further study is needed to understand whether this wide range of gut microbial dysbiosis and metabolite alterations impact clinical outcomes and if they can be used as predictive biomarkers or manipulated to improve transplant outcomes.

## Introduction

The human gut microbiome is composed of over a thousand microbial species that reside within the lumen of the gastrointestinal tract (1, 2). Recent metagenomic and metabolomic analyses have revealed that interactions between microbes and their human hosts may have far-reaching impacts on human health (3, 4). Medical interventions, in particular antibiotic administration, can lead to loss or suppression of beneficial commensal microbes and their associated metabolites, resulting in unstable “dysbiotic” states.

Studies in hematopoietic stem cell (HSCT), kidney, and liver transplantation have shown associations between dysbiosis and adverse outcomes, including allograft rejection and death (5–8). Fewer studies have examined the role of the gut microbiome in heart transplantation (HT) outcomes. In pre-clinical murine models, changes to the gut microbiome have an impact on cardiac allograft survival (9–11). Prior studies have shown reduced gut microbial diversity among patients with heart failure (12–14). Furthermore, gut microbiome diversity decreases with worsening heart failure and persists even after HT (15).

The mechanism for this may be through the action of gut microbial metabolites on recipient immune systems. In vitro and animal studies have utilized metabolomic analysis to demonstrate how gut microbial-derived short chain fatty acids (SCFA) and secondary bile acids may be immunomodulatory, affecting post-transplant outcomes (16–21). Such metabolomic studies are limited in humans. Instead, human studies quantify butyrate-producing bacteria or the presence of genes that encode for metabolite production pathways and associate these to post-transplant clinical outcomes (8, 22–24). In so doing, such analyses link microbial metabolites to clinical outcomes without directly quantifying stool microbial metabolite concentrations. Only in limited studies of HSCT recipients have stool concentrations of gut microbial metabolites been directly measured with quantifiable results and correlated with adverse outcomes such as graft-vs.-host disease (25, 26).

To date, no studies have examined gut microbial production of immunomodulatory metabolites at the time of HT, when the recipient immune system is first introduced to alloantigens present in the cardiac allograft. To begin to understand the potential influence of the gut microbiome on alloimmunity during this crucial period, we aimed to characterize the gut microbiome and its metabolite production in the peri-HT period. We hypothesize that, compared to healthy donors, HT recipients will demonstrate gut dysbiosis and that such dysbiosis will result in reduced production of key, potentially immunomodulatory, gut microbial metabolites.

## Materials and methods

### Participants

This was a prospective cohort study of adult HT recipients at a single institution from July 2020 to February 2021. Inclusion criteria were age ≥18 years, ability to provide informed consent, and active listing for HT, including multi-organ transplantation (heart-kidney, heart-liver, and heart-liver-kidney). Subjects were excluded if they were <18 years old, unable to consent, follow for ≥2 years, or provide a stool sample within 14 days of HT. A cohort of healthy donors were also recruited through the Duchossois Family Institute at the University of Chicago. The study was approved by the Institutional Review Board at our institution. Written informed consent was obtained from all participants.

### Clinical data collection

Demographic and clinical data were collected through review of the medical record. These included particular factors that could impact the gut microbiome such as length of hospitalization prior to sample collection and antibiotic exposures in the 3 months pre-HT.

### Specimen collection and processing

Fecal samples were collected at time of study enrollment from healthy donors and HT recipients. When able to be produced by the HT subjects, samples were collected within 14 days pre- and/or post-HT.

To prevent contamination, aseptic conditions were maintained during fecal sample aliquoting and collection. Samples were immediately stored at −80°C post collection and freeze-thaw cycles were avoided to conserve microbiome diversity and prevent contamination. A unilateral workflow was maintained through designated laboratory areas for pre- and post-PCR processing. Testing with appropriate negative controls was conducted to evaluate for contamination from the reagents used for library preparation.

### Metagenomic analyses

Fecal samples underwent next generation shot-gun DNA sequencing. To minimize biases and optimize yield of both gram-positive and gram-negative organisms (27), mechanical disruptions with a bead beater (BioSpec Product) were conducted and samples were further purified with QIAamp mini spin columns (Qiagen). Enzymatic fragmentation during library preparation ensured consistent fragment lengths and PCR-free protocols reduced biases introduced by PCR cycles. Robust libraries were generated with 200 ng DNA input using a PCR-free DNA sequence kit (QiaSeq FX DNA library kit, Qiagen).

Inputs of starting material were normalized at every step of the workflow to reduce sampling size bias. Purified DNA was quantified with a Qubit 2.0 fluorometer. DNA input for library preparation were kept consistent at 200 ng. Prior to sequencing, libraries were quantified, their sizes were determined, and pooled at equimolar concentrations to ensure even read distribution across all samples.

Samples were then sequenced on the Illumina HiSeq platform, producing around 7–8 million PE reads per sample with read length of 150 bp. Adapters were trimmed off from the raw reads, and their quality was assessed and controlled using Trimmomatic (v.0.39) (28), then human genome was removed by kneaddata (v0.7.10, https://github.com/biobakery/kneaddata). Taxonomy was profiled using metaphlan4 (29).

All aspects of the next-generation sequencing workflow were automated to increase replicability and consistency across samples. Random spot checks of previously sequenced samples were conducted to ensure taxonomic profile consistency and validate data reproducibility.

### Metabolomic analyses

Short chain fatty acids (SCFA; butyrate, acetate, propionate, succinate) were derivatized with pentafluorobenzyl bromide and analyzed via negative ion collision induced-gas chromatography-mass spectrometry (Agilent 8,890). Eight bile acids [primary: cholic acid; conjugated primary: glycocholic acid, taurocholic acid; secondary: deoxycholic acid, lithocholic acid (LCA), isodeoxycholic acid; modified secondary: alloisolithocholic acid (alloisoLCA) and 3-oxolithocholic acid (3-oxoLCA)] were quantified (µg/ml) by negative mode liquid chromatography-electrospray ionization-quadrupole time-of-flight-MS (Agilent 6,546).

### Statistical analysis

Statistical analysis was conducted using R statistical language (v4.1.1). Continuous variables were compared using Wilcoxon rank-sum test, and *p*-values were adjusted for multiple comparisons by following the Benjamini-Hochberg method, as the majority of variables were not normally distributed. Categorical variables were compared using Chi-Square/Fisher’s Exact test. Kendall rank correlations were performed to determine associations between measured metabolites.

Alpha-diversity (a reflection of the number of unique bacterial taxa and their relative abundances) of fecal samples was estimated using Inverse Simpson Index.

Beta-diversity (compositional similarity between cohorts) analysis was performed with Uniform Manifold Approximation and Projection (UMAP), and difference in Bray-Curtis distances between groups were tested by Permutational Multivariate Analysis of Variance (PERMANOVA).

The linear discriminant analysis effect size (LEfSe) method was utilized to identify bacterial taxa more abundant within one cohort compared to another (30). The LEfSe algorithm uses the non-parametric Kruskal Wallis statistical test to compare all taxa at different taxonomic levels between groups, and paired Wilcoxon Rank Sum to test among subgroups. It then builds a linear discriminant analysis (LDA) model which utilizes continuous independent variables (e.g., bacterial abundance) to predict one dependent variable (e.g., healthy donors vs. HT recipients) and provides an effect size for the significantly different taxa (30). Higher LEfSe indicates that the dependent variable (e.g., healthy donors) has increased abundance of that specific microbial species compared to the other dependent variables (e.g., HT recipients).

To investigate the impact of clinical factors to the gut microbiome, additional analyses were conducted on HT recipients stratified based on transplant type (single vs. multi-organ), tertiles of alpha-diversity, and butyrate and bile acid production. Further analysis was conducted based on median pre-sample hospital length of stay and antibiotic exposure to high impact antibiotics within 3 months pre-HT. Adjusted *p*-values ≤0.05 were considered significant.

## Results

58 HT recipients were enrolled in the study, of whom 48 (33 HT, 15 multi-organ transplants) were able to produce stool samples for analysis. Demographic and clinical characteristics were compared to those of 20 healthy donors (Table 1). HT recipients were significantly older [57 (20–71) vs. 31.5 (18–63) years, *p* < 0.001], and were more likely to be male or black compared to healthy donors (Table 1).

**Table 1:** Cohort demographic and clinical characteristics.

	Healthy donor (*n* = 20)	Heart transplant (*n* = 48)	*p*
Age (median, min-max)	34.5 (18–63)	57 (20–71)	0.0001
Sex			0.002
Male	7 (35%)	36 (75%)	
Female	13 (65%)	12 (25%)	
Ethnicity			0.027
African American	1 (5%)	20 (42%)	
Asian	3 (15%)	3 (6%)	
Hispanic	3 (15%)	5 (10%)	
White	13 (65%)	20 (42%)	
Chicago resident	20 (100%)	41 (85%)	
Co-Morbidities			
Prior smoker		17 (35%)	
Hypertension		34 (71%)	
Diabetes		14 (29%)	
Autoimmune disease		1 (2%)	
Prior pregnancy		10 (21%)	
Prior blood transfusion		18 (38%)	
History of immunosuppression		11 (23%)	
Etiology of heart failure			
ICM		9 (19%)	
NICM		39 (81%)	
Pre-transplant support			
Mechanical circulatory support		37 (77)	
LVAD ^a^		9 (18)	
Inotropes		34 (71)	
Type of transplant			
Heart		33 (69%)	
Heart-kidney		11 (23%)	
Heart-liver		2 (4%)	
Heart-liver-kidney		2 (4%)	
Immunosuppression			
Basiliximab		41 (85%)	
Tacrolimus		48 (100%)	
Methylprednisolone, IV		48 (100%)	
Mycophenolate mofetil		48 (100%)	
Other medications			
Warfarin		24 (50%)	

^a^ LVAD, Left ventricular assist device e.g. HeartMate 2, HeartMate 3, Heartware HVAD.

Clinical co-morbidities were only available for the HT cohort (Table 1), with the most common etiology of heart failure being non-ischemic cardiomyopathy (81%). The median pre-HT hospital length-of-stay was 16 days (range = 0–62 days). Thirty-seven patients (77%) were supported with mechanical circulatory support devices and 34 (71%) were on intravenous continuous inotropes prior to HT (Supplementary Table S1).

**Figure 1:**
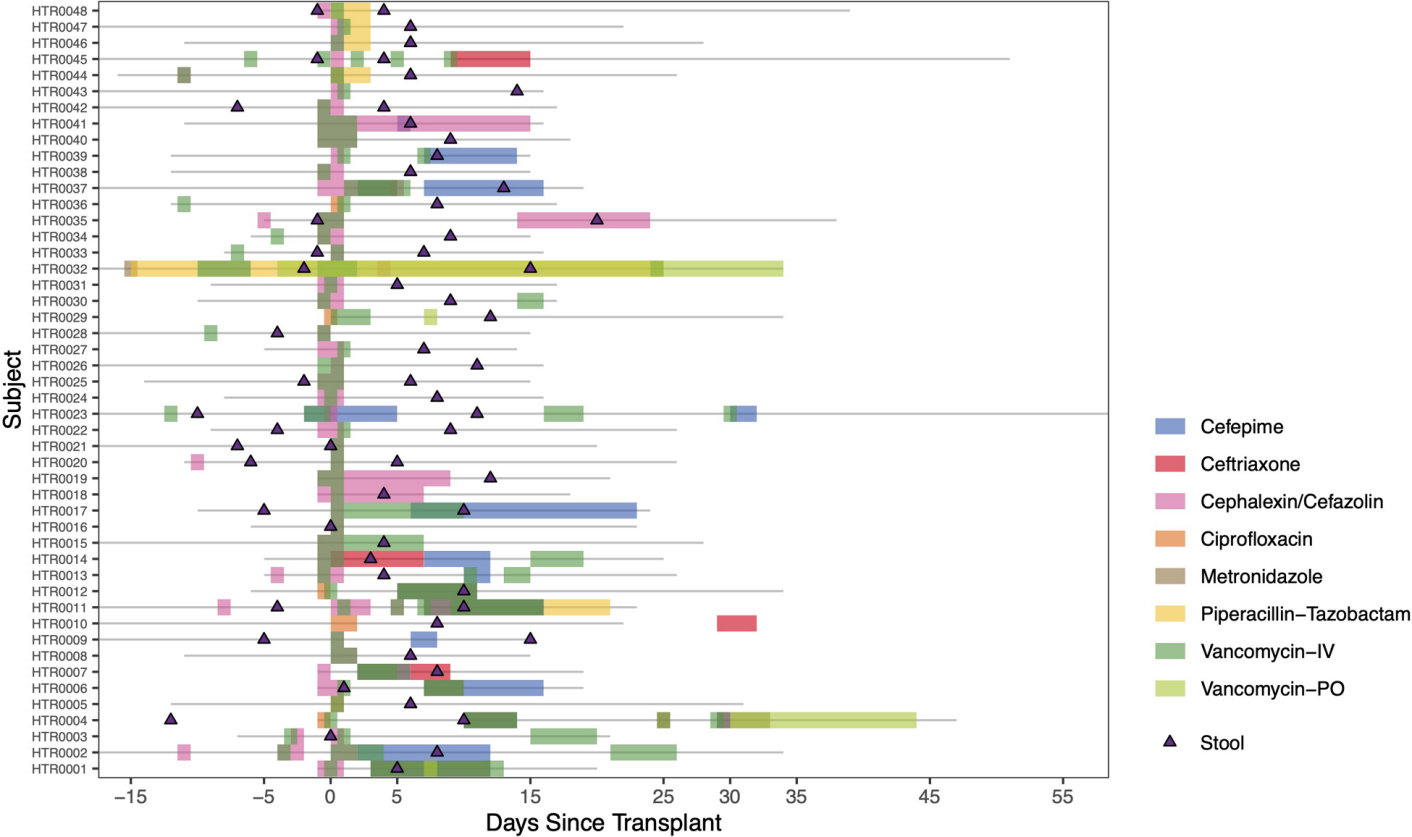
Heart transplant timelines. Individual timelines of admission duration (grey lines) and peri- and post-transplant antibiotic administration in reference to day of transplantation (Day 0) are shown here. Triangles indicate date of stool sample collections. Separate colors indicate notable antibiotic courses received during this timeframe.

Twenty-five (52%) of HT recipients received antibiotic therapy within 3 months pre-HT (Supplementary Table S2). All HT recipients received prophylactic intravenous antibiotics per protocol, including cefazolin (unless already receiving another therapeutic dose of cephalosporin) and vancomycin (Figure 1).

Peri-operatively, 85% of HT recipients received induction with basiliximab, and 100% received high dose steroid therapy with methylprednisolone and anti-metabolite therapy with mycophenolate mofetil (Table 1). Calcineurin inhibitor therapy with tacrolimus was initiated within 24–72 h following transplantation at the discretion of the clinical team.

### The Gut microbiome of heart transplant recipients vs. healthy donors

**Figure 2:**
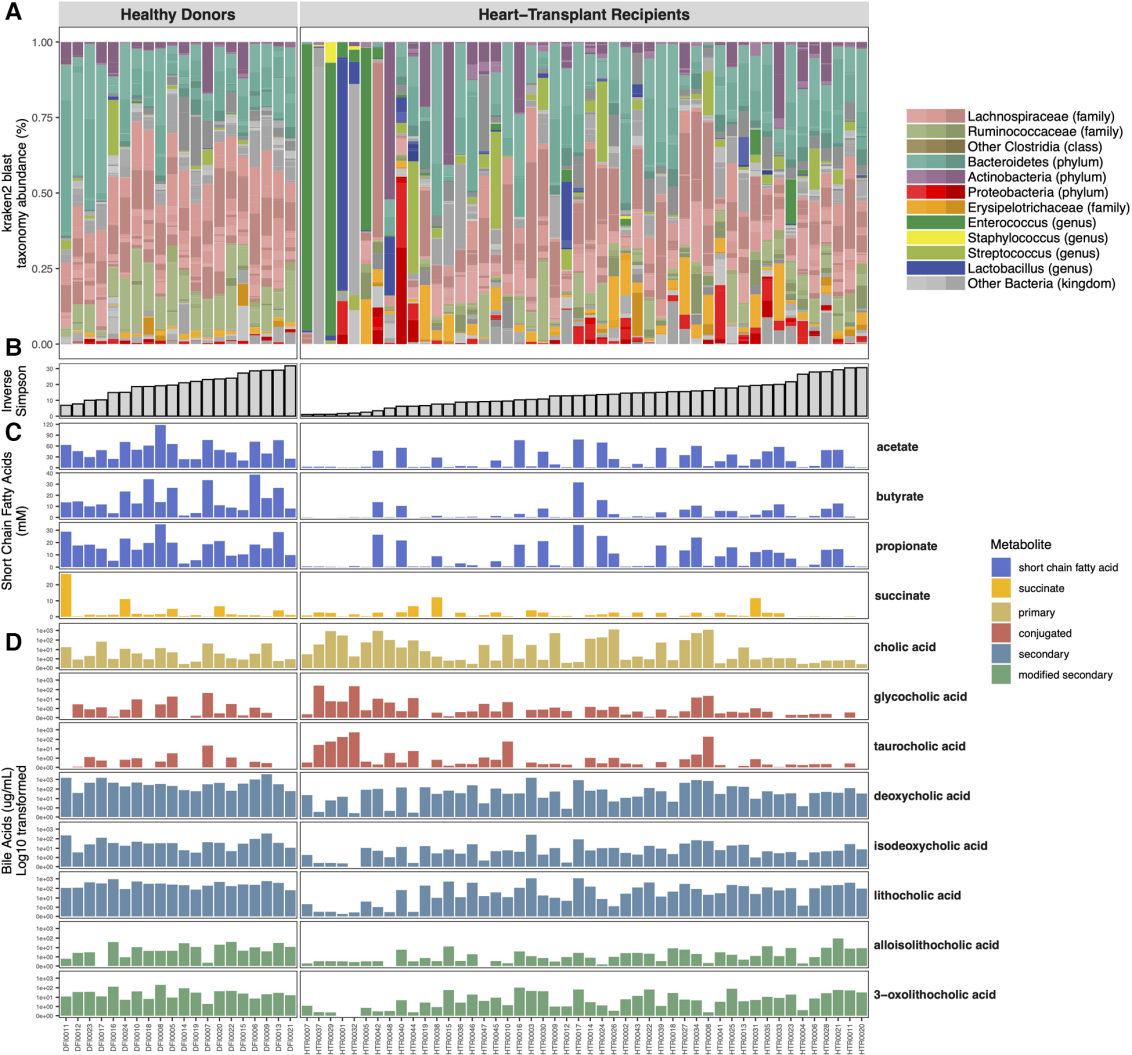
Shotgun metagenomic and metabolomic analysis of the microbiome. Vertical columns represent individual subjects and metagenomic sequencing data is color-coded with the relative abundance of specific bacterial taxa comprising their microbiome (**A**). Subjects are organized in ascending order of within-sample (alpha) diversity (**B**). Mass-spectrometry quantification of gut microbial metabolite levels demonstrates variable concentrations of bile acids (**C**) and short chain fatty acids within the fecal samples (**D**).

Each participant’s gut microbiome composition is depicted in Figure 2. Similar to previously published data on the normal human gut microbiome (3), the gut microbiome of healthy donors is diverse with a high relative abundance of commensal anaerobic bacterial taxa belonging to the phyla Bacteroidetes and Firmicutes*,* including the families *Ruminococcaceae* and *Lachnospiraceae* of the class Clostridia.

**Figure 3:**
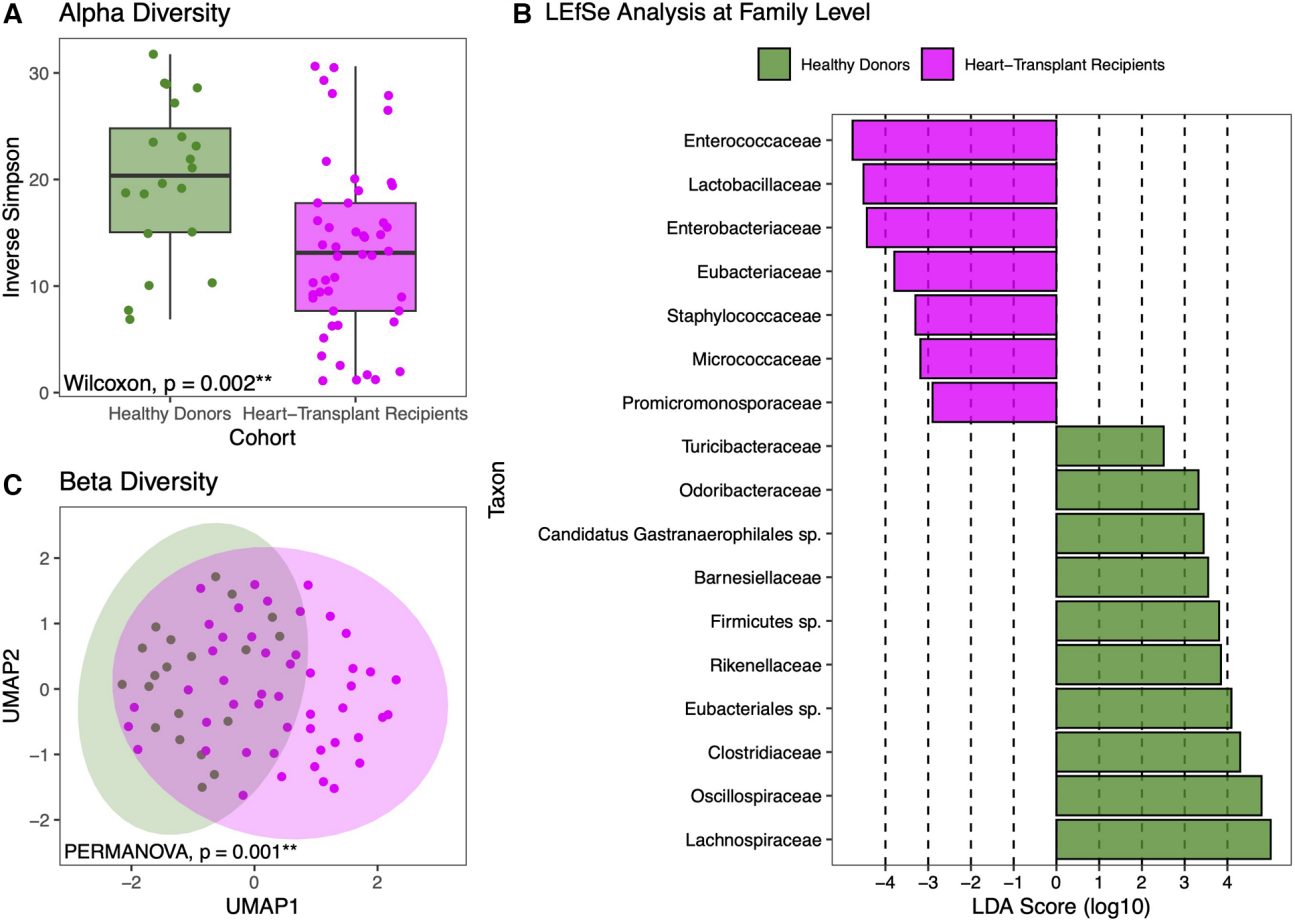
Diversity and compositional analysis. Healthy donors have more diversity in their individual samples than heart transplant recipients (**A**). LEfSe analysis showing statistically significant differences between the two cohorts at the family level (**B**). UMAP-based beta-diversity analysis further demonstrating that heart transplant recipients have gut microbiomes that are more compositionally similar to each other than to that of healthy donors, even after adjustment for age, sex, and race (**C**).

HT recipients also exhibit a wide range of gut microbial compositions. Many express a similar abundance of commensal anaerobic bacteria found among healthy donors. However, the microbiomes of some HT recipients were marked by loss of commensal anaerobic bacteria and consequent expansion of 1 or 2 bacterial genera, including *Enterococcus*, *Enterocloster*, *Ligilactobacillus*, *Escherichia*, and *Klebsiella* (Figure 2A). The gut dysbiosis among HT recipients is reflected in the significantly reduced alpha diversity in this cohort compared to healthy donors (Inverse Simpson 14.42 vs. 18.86, *p* = 0.05) (Figure 3A).

LEfSe analysis revealed that at the phylogenetic family level, HT recipients had higher abundances of *Enterococcaceae, Lactobacillaceae, Enterobacteriaceae, Staphylococcaceae, Micrococcaceae*, and *Promicromonosporaceae* (Figure 3B). To examine compositional differences between HT recipients and healthy donors, beta-diversity analysis was performed and revealed distinct clustering, even after adjusting for age, sex, and race, (PERMANOVA *p* = 0.001) (Figure 3C) indicating that the gut microbiome compositions of healthy donors are more similar to each other than to that of HT recipients.

### Metabolite analysis: SCFA and bile acids

**Figure 4:**
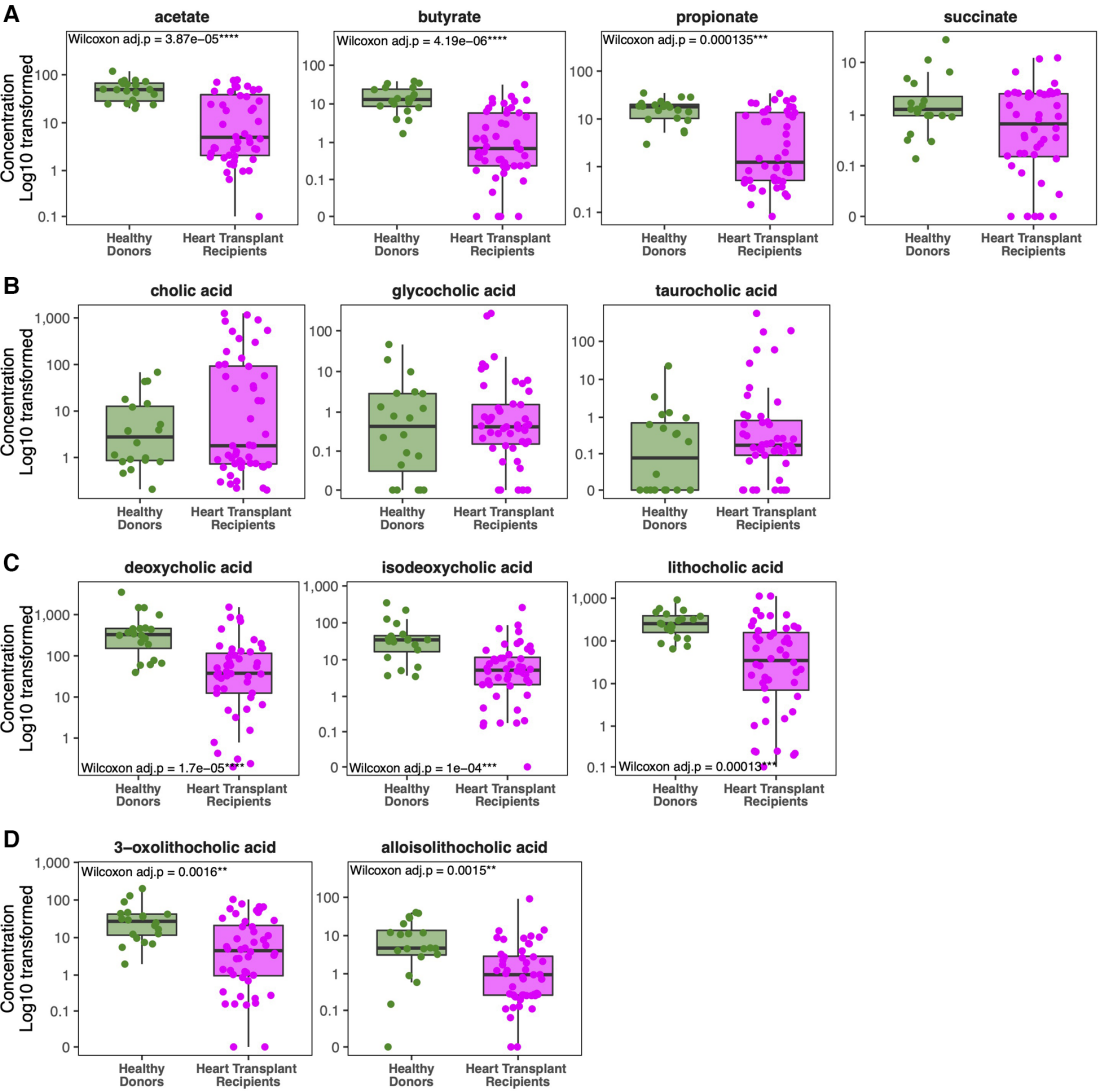
Comparison of metabolite concentrations between cohorts. Heart transplant recipients exhibit lower concentrations of short chain fatty acids (**A**) compared to healthy donors. Although they exhibit similar levels of primary and conjugated primary bile acids (**B**), heart transplant recipients exhibit lower levels of secondary bile acids (**C**) and modified secondary bile acids (**D**) compared to healthy donors.

Healthy donors exhibit marked differences in metabolite concentrations compared to HT recipients. Whereas healthy donors produce similar levels of SCFA between individuals, we observed a wide range of fecal SCFA concentrations among HT recipients (Figure 4A). HT recipients produce significantly lower median levels of fecal butyrate (0.695 vs. 13.04 mM, *p* < 0.001), acetate (4.98 vs. 48.98 mM, *p* < 0.001), and propionate (1.26 vs. 17.95 mM, *p* < 0.001) compared to healthy donors (Figure 4A). Consistent with previously published studies, individuals who express greater loss of normal commensal bacteria and domination with 1–2 bacterial taxa produce lower concentrations of fecal SCFA (31–34).

While healthy donors generally have similar concentrations of fecal bile acids across individuals, there is significant inter-individual variability in secondary bile acid concentrations among HT recipients, as seen with SCFAs (Figure 4C–D). Patients with microbiota domination by non-obligate anaerobes have lower secondary bile acid concentrations. While concentrations of host-derived primary and conjugated bile acids are similar between HT recipients and healthy donors (Figure 4B), HT recipients have significantly lower median levels of the secondary bile acid LCA (34.99 vs. 254.01 *μ*g/ml, *p* = 0.003), deoxycholic acid (37.80 vs. 327.50 *μ*g/ml, *p* < 0.001), and isodeoxycholic acid (5.21 vs. 34.52 *μ*g/ml, *p* = 0.002) compared to healthy donors (Figure 4C). Modified from LCA, alloisoLCA (0.95 vs. 4.71 *μ*g/ml, *p* = 0.036) and 3-oxoLCA (4.51 vs. 27.20 *μ*g/ml, *p* = 0.032) were also significantly lower among HT recipients compared to healthy donors (Figure 4D).

**Figure 5:**
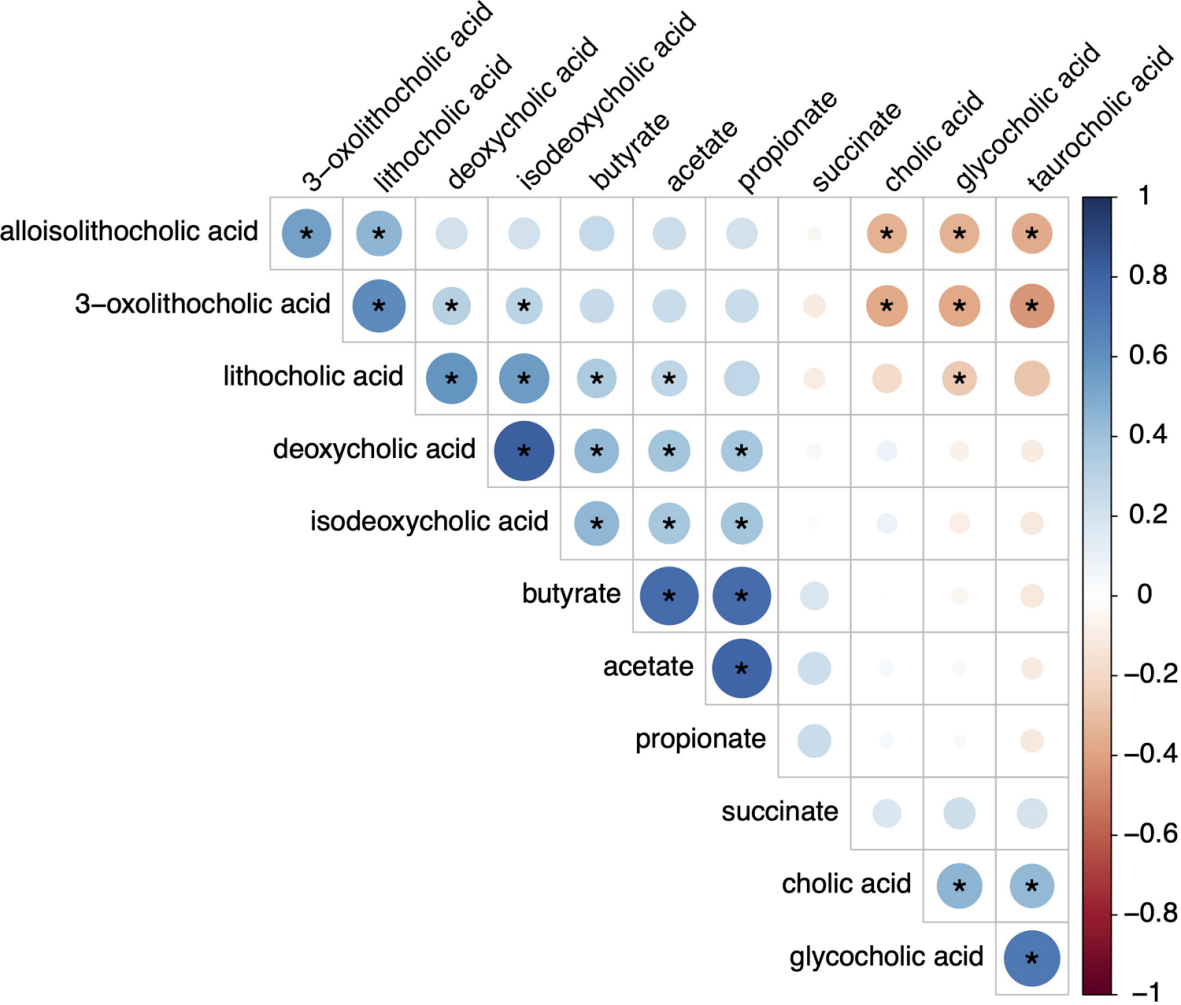
Spearman correlations of metabolites. This plot demonstrates correlations between the short chain fatty acids and bile acids. Blue indicates positive correlations and red indicates negative correlations. Deeper hues indicate stronger correlations with the (*) indicating significance.

To determine whether there is concordance between SCFA and bile acid producers, Spearman correlations were performed between SCFA and bile acid levels (Figure 5). Although there are strong correlations between the concentrations of individual SCFA to other SCFA as well as secondary bile acids to other bile acids, SCFA are only moderately correlated to levels of either secondary or modified secondary bile acids. This suggests that factors resulting in elevated SCFA and bile acid levels are likely independent of each other.

### Association with clinical factors

To examine the potential clinical drivers of these compositional and metabolite differences, the HT cohort was divided into tertiles based on (1) inverse Simpson index, (2) butyrate production, (3) secondary bile acid production, and (4) modified secondary bile acid production. When divided into tertiles of low, intermediate, or high inverse Simpson index, there were no differences between groups in terms of age, sex, race, comorbidities, heart failure etiology, or other clinical factors (Supplementary Figure S1). Similarly, no differences were found between tertiles of secondary and modified secondary bile acid production. Analysis of butyrate revealed no association to demographic and comorbid factors.

To analyze the potential impacts of prolonged hospitalization prior to sample collection, a separate subgroup analysis based on median pre-sample length-of-stay was conducted. Subjects who experienced short hospitalization [median = 10, IQR (6, 14) days] were compared to long hospitalization [median = 22, IQR (18, 32) days]. There were no significant differences between the groups in terms of alpha or beta diversity (Supplementary Figure S2). Subjects with a long pre-HT hospitalization had significantly lower levels of LCA (67.6 vs. 251.0, *p* = 0.02) but exhibited no differences in SCFA and other secondary bile acid levels (Supplementary Figure S3).

To understand the impact of antibiotics on the gut microbiome, antibiotic exposures 3 months pre-sample were also analyzed (Supplementary Table S2). 52 of the 58 HT subjects were exposed to antibiotics within 7 days pre-sample and, thus, we focused our analysis on this timeframe. There were no significant differences in gut microbial diversity or metabolite production between HT recipients when exposures to all antibiotics were considered, 7 days pre-sample collection (Supplementary Figures S4, 5).

**Figure 6:**
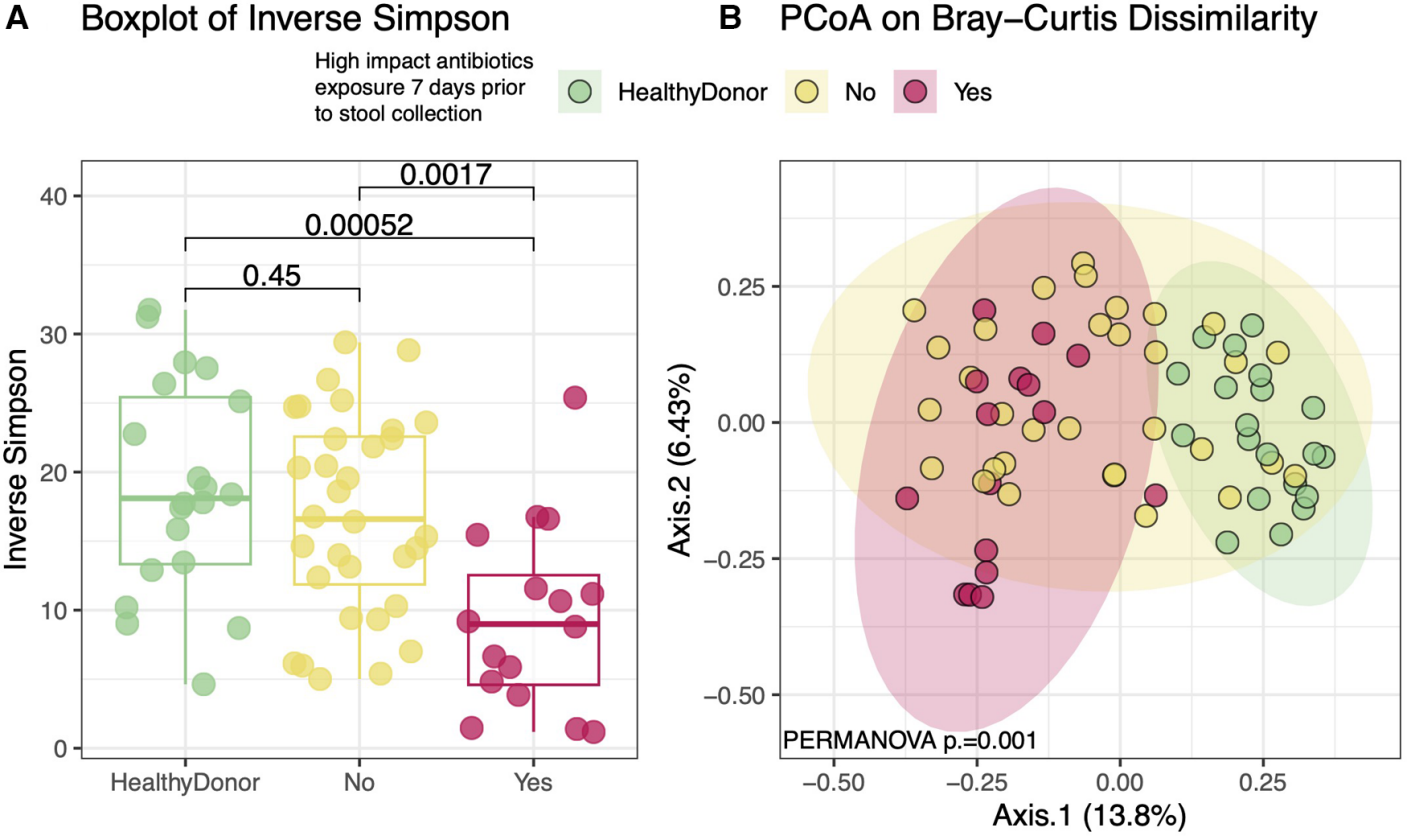
Diversity and compositional analysis between “high impact” vs No significant antibiotic exposure 7 days Pre-sample collection. Heart transplant recipients who were exposed to high impact antibiotics had significantly lower within-sample diversity when compared to either healthy donors or transplant recipients who were not exposed to such antibiotics (**A**). Heart transplant recipients who received any of the high impact antibiotics appear to have compositionally similar gut microbiomes that distinguish them from either healthy donors or those transplant recipients who did not receive these antibiotics (**B**). Of note, heart transplant recipients who were not exposed to these antibiotics were also more compositionally similar to each other than healthy donors (*p* = 0.001). *High impact antibiotics = oral vancomycin, cefepime, piperacillin/tazobactam, metronidazole, and ciprofloxacin*.

**Figure 7:**
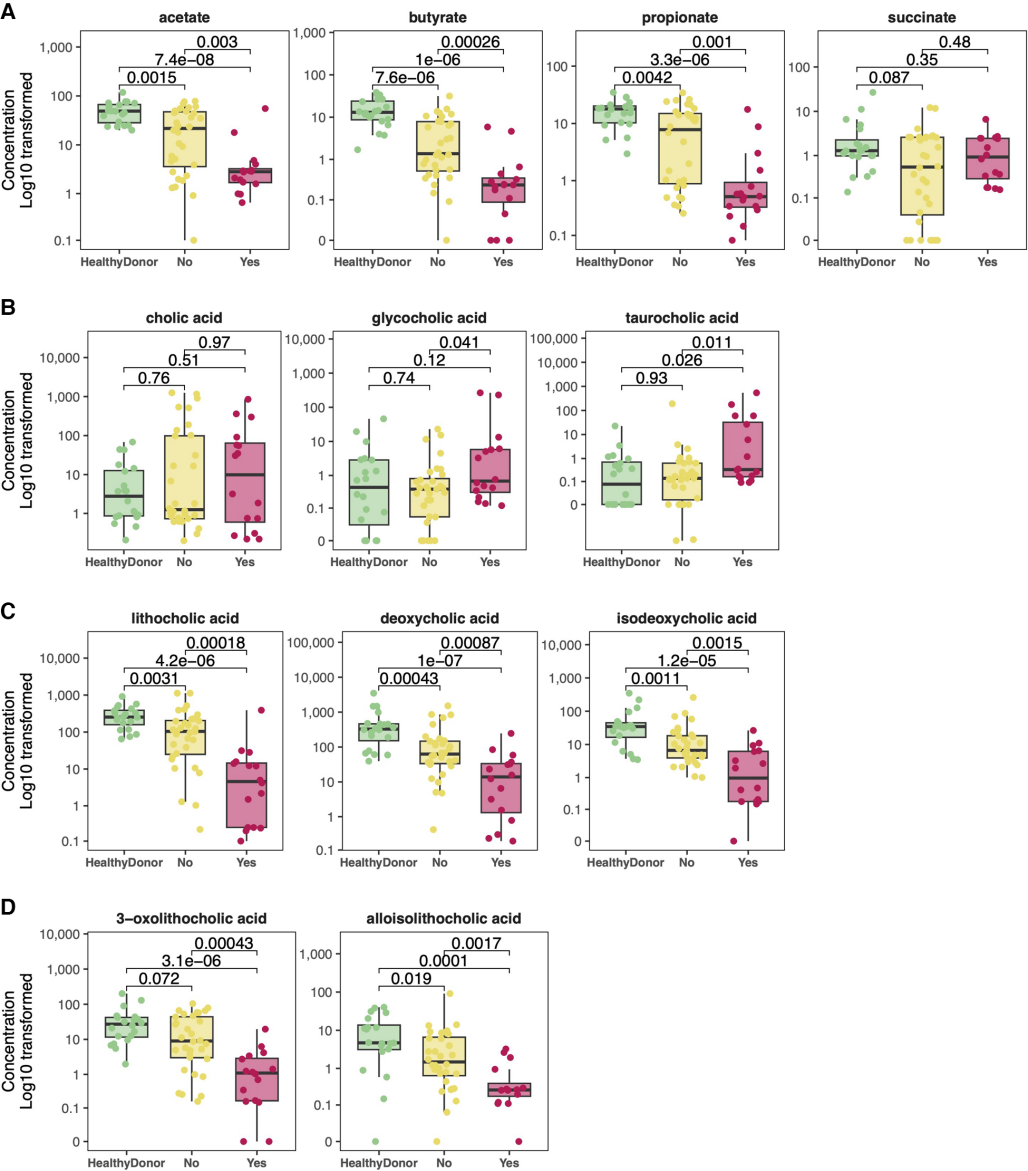
Comparison of metabolite concentrations by high impact antibiotic exposure. Significantly lower levels of short chain fatty acids (**A**), as well as secondary (**C**), and modified secondary bile acids (**D**) were noted amongst the recipients who had been exposed to high impact antibiotics as compared to either healthy donors or to those recipients who had not been exposed.

However, previous studies have demonstrated that certain “high impact” antibiotics drastically reduce the concentrations of healthy commensal anaerobic gut microbes (35–38). Of these, oral vancomycin, cefepime, piperacillin/tazobactam, metronidazole, and ciprofloxacin were observed in our cohort. 16 HT subjects were treated with one or more of these high impact antibiotics in the 7 days prior to sample production. These subjects had significantly lower within sample diversity (Figure 6A) compared to healthy donors and those who had not been exposed to these specific antibiotics (*p* = 0.0014). These subjects also had gut microbial compositions that were more similar to each other than to those who were not exposed to these antibiotics (Figure 6B, *p* = 0.001). The “high impact” antibiotic cohort consistently demonstrated significantly reduced metabolite levels of SCFA and all secondary and modified secondary bile acids, especially when compared to healthy donors (Figure 7).

### Pre- and post-heart transplant analysis

To analyze the impact of the peri-HT period on the gut microbiome, sequential pre- and post-HT samples from the same HT recipients were compared. Successful pre- and post-HT stool collection was achieved for 17 HT recipients, a median of 5 days before and 10 days after HT (Figure 1). There was no difference in alpha or beta diversity between pre- and post-HT samples (Supplementary Figure S6). There were also no significant differences in metabolite production between these two timepoints (Supplementary Figure S7).

### Single vs. multi-organ analysis

**Figure 8:**
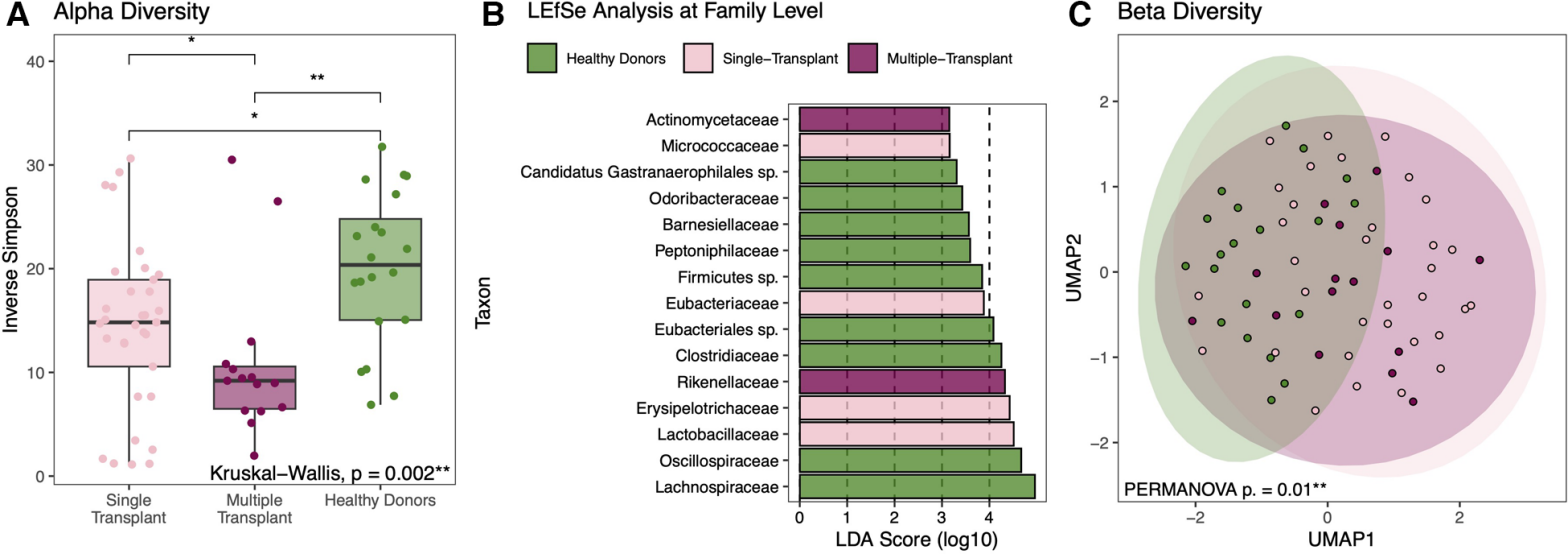
Diversity and compositional analysis between heart alone vs multi-organ transplant vs healthy donors. Although not significantly different when compared to heart-alone recipients, within-sample diversity is significantly lowest among multi-organ transplant recipients compared to healthy donors (**A**). Each group is marked by increased abundance of specific bacterial taxa with the phylogenetic family level selectively shown here (**B**). When comparing composition across groups, there is a significant degree of compositional differences between healthy donors and either transplant group but not between the single or multi-organ transplant recipients (**C**).

Given the possible differences in comorbidities, illness severity, and antibiotic exposure, the entire cohort was stratified based on single vs. multi-organ transplant (11 heart-kidney, 2 heart-liver, and 2 heart-liver-kidney). Compared to the 33 heart-alone recipients, multi-organ transplant recipients had a similarly wide variability in gut microbial composition with a wide spectrum of alpha-diversity. Within-sample diversity was significantly different between all groups (Figure 6A). Multi-organ transplant recipients had the lowest alpha-diversity compared to healthy donors (10.9 vs. 20.0, *p* = 0.0012; Figure 8A). The three subgroups each had significantly increased abundance of specific bacterial taxa (Figure 8B). Metabolite analysis showed that SCFA and secondary bile acid production was overall similar between the single and multi-organ transplant recipients but remained lower than that of healthy donors (Supplementary Figure S8). Beta-diversity analysis showed that there was a significant difference in gut microbial composition between healthy donors and HT recipients but no significant difference between single and multi-organ transplant recipients (Figure 8C).

## Discussion

This study is the first to characterize the gut microbiome along with its bioactive byproducts among heart and multi-organ transplant recipients at the time of transplantation. To our knowledge, this is the first report directly measuring stool metabolites amongst solid organ transplant recipients. Our analysis adds to the growing body of evidence that, compared to healthy donors, the gut microbiome of HT recipients is marked by reduced within-sample diversity and increased dysbiosis. Such dysbiosis results in lower stool concentrations of key immunomodulatory gut microbial metabolites such as SCFA and secondary bile acids, when compared to levels in healthy donors. There is also a notable degree of inter-individual variability in gut microbial diversity that appears driven by heart failure illness severity and exposure to certain high impact antibiotics.

### Gut microbial diversity in heart transplant recipients

Heart failure has been associated with reduced within-sample or alpha diversity in a number of cohort studies (15, 39). The degree of dysbiosis appears to worsen with worsening heart failure (15, 40). Having the most severe forms of heart failure illness, our HT recipients unsurprisingly demonstrate significantly dysbiotic gut microbiomes. Overall disease severity appears to further these patterns as gut microbial dysbiosis appears particularly pronounced among multi-organ transplant recipients. Demographic factors and other comorbidities do not appear to impact this variability, contrary to what has been previously described (41). Nor do they appear to be significantly associated with pre-HT hospitalization length or the interventions administered in the peri-HT period.

It is likely that beyond illness severity, a primary driver of gut dysbiosis among HT recipients is exposure to high impact antibiotics. We demonstrate that even in the absence of such exposures, HT recipients have significantly reduced gut microbial diversity and metabolite production. Exposure to specific high impact antibiotics exacerbate these trends. As most of the exposures to antibiotics occurred within 7 days of sample production in our cohort, the duration with which these alterations persist warrants further study. Prior studies among healthy individuals have demonstrated high inter-individual variability in gut microbial recovery rates after antibiotic discontinuation, with some individuals experiencing changes that persist for months (38, 42). Whether HT recipients who have baseline gut dysbiosis are able to achieve similar levels of gut microbial recovery after exposure to high impact antibiotics remains unclear. Most importantly, whether these observations ultimately impact clinical HT outcomes through a gut microbial-dependent mechanism warrants further investigation.

Compared to healthy donors, our HT recipients have reduced alpha-diversity, partially due to significantly lower abundance of the phylum Firmicutes, including the species *F. prausnitzii*. *F. prausnitzii* serves as a marker of gut microbial health (43) and exerts anti-inflammatory effects, the loss of which may lead to a variety of inflammatory disease states (44–47). In post-liver transplant patients, low *F. prausnitzii* levels have been observed (48).

The reduced abundance of commensal anaerobic bacteria that we observe amongst HT recipients compared to healthy donors often coincides with the expansion of certain bacterial taxa, including known pathogens. In other forms of transplantation, similar patterns result in significant clinical outcomes. In allo-HSCT recipients, higher levels of *Enterobacteriaceae* were correlated to increased mortality (49). In one kidney transplant cohort, acute rejection within 90 days of transplantation was associated with higher relative abundance of *Lactobacillales* and *Enterococcus* (7). Similarly, liver transplant recipients with acute cellular rejection had increased abundance of *Enterobacteriaceae, Streptococcaceae*, and *Bifidobacteriaeceae* (6)*.* Further study is needed to understand whether the strikingly similar patterns of dysbiosis among our HT recipients could result in similar clinical correlations.

### Gut microbiome metabolites and alloimmunity

The link between gut dysbiosis and transplantation outcomes may stem from the increasingly evident role of the gut microbiome on regulatory T cell (Treg) differentiation. Tregs may promote allograft tolerance by suppressing immune responses leading to rejection (50). Specific commensal species e.g., *Clostridia* (51, 52) and *F. prausnitzii* (44) or the presence of a stereotypical commensal anaerobic bacterial community (53, 54) appear to induce Treg maturation, likely through the impact of the metabolites they produce (55).

An important class of gut-derived immunomodulatory metabolites, SCFA are produced as gut microbes ferment dietary fiber. Of these, butyrate has been associated with reduced inflammation and oxidative stress (56) and increased Treg maturation (17). In mouse models of HSCT, increased butyrate has been associated with increased Treg populations (21). Exogenous butyrate administration in mice has been shown to mitigate graft-vs.-host disease in HSCT (21) and increase renal allograft survival, likely through a Treg-dependent mechanism (18). In our study, HT recipients have reduced abundance of *Roseburia*, *Ruminococcus*, and *F. prausnitzii*, some of the most potent butyrate-producing bacteria in the human colon (57). Consequently, we observe a wide range of butyrate production amongst our HT recipients, with many producing significantly less than healthy donors.

Bile acids have also emerged as an important mediator of gut microbial influence on adaptive immunity. Synthesized from cholesterol in the liver and then secreted into the gut to aid in digestion (58), primary bile acids that evade enterohepatic recirculation are metabolized solely by gut microbes into secondary bile acids and their derivatives (59, 60). Secondary bile acids such as LCA, and its derivatives 3-oxoLCA and isoalloLCA, have been shown to promote differentiation of Tregs (19). Hang et al. demonstrated that exogenous administration of 3-oxoLCA and isoalloLCA to mice increased Treg differentiation (19). Compared to healthy donors, our HT recipients had lower abundance of *Clostridium spp,* one of the main genera responsible for the modification of primary into secondary bile acids (61), and had lower levels of secondary bile acids.

Our study adds to the limited data demonstrating reduced gut microbial SCFA among patients with heart failure (62). We are also the first to describe reduced secondary bile acid concentrations in stool samples of patients with chronic heart failure. In the only other study directly measuring bile acids in chronic heart failure, Mayerhofer et al. demonstrated the opposite, citing an elevation of secondary bile acids in this population (63). The reasons for and implications of this discrepancy are unclear.

To our knowledge, direct measurements of stool gut microbial metabolite concentrations have not previously been performed among human solid organ transplant recipients. Without metabolomic analysis, prior studies in kidney and liver transplantation have inferred the impact of gut-derived metabolites based on surrogates such as quantification of known butyrate-producing bacteria or presence of genomic sequences with the potential to encode known metabolic pathways. In contrast, limited direct analyses of stool metabolite concentrations have been performed among HSCT recipients. Among HSCT, lower butyrate stool concentrations have been correlated to an increased risk for graft-vs.-host disease (26) and higher stool butyrate concentrations to an 80% reduction in lower respiratory tract infections (25). These studies highlight how metabolomic analyses may further our mechanistic understanding of the influence of the gut microbiome on outcomes of HT recipients.

Additionally, elevated SCFA and secondary bile acid levels are likely independent of each other. We found only moderate correlations between SCFA and secondary or their derived bile acid levels, consistent with previously published research indicating that the bacterial taxa responsible for producing SCFA or secondary bile acids are distinct (31, 33, 59). This may also be due to inter-individual differences in fiber intake or cholesterol metabolism. Understanding the gut microbial characteristics that can generate higher concentrations of these potentially beneficial metabolites may lead to novel treatments for transplant recipients.

Our results should be interpreted with significant limitations in mind. First, the small cohort size at a single center may limit its generalizability to other patient populations. Second, the limited follow-up period did not allow for exploration of the impact of the findings on clinical outcomes such as rejection or mortality. Further, we were not able to capture granular health-related comorbidities amongst our healthy donors. Potentially and most importantly, we were unable to account for the impact of health-related behaviors such as diet and other lifestyle factors on the gut microbiome. We aim to address these and many other issues with further recruitment for future analyses from this study.

In conclusion, we demonstrate that HT recipients have patterns of gut dysbiosis and metabolite production that distinguish them from healthy donors. They also have a significant degree of inter-individual variability in gut microbial composition which result in marked differences in measurable concentrations of metabolites. Many of these patterns have been associated with immune consequences that have been linked to adverse outcomes in other transplant populations. Larger and more longitudinal studies are necessary to understand the true nature of the interactions between the gut microbiome, its metabolites, and outcomes in HT. Gaining this knowledge may present a unique modality to improve the care of HT recipients.

## Data Availability

The datasets presented in this study can be found in online repositories. The names of the repository/repositories and accession number(s) can be found below: BioProject Number: PRJNA845905.
