# Supplementary material for: Reduced immunomodulatory metabolite concentrations in peri-transplant fecal samples from heart allograft recipients
Source: Front Transplant. 2023 Jul 17;2:1182534. doi: 10.3389/frtra.2023.1182534 (PMC11235359; doi:10.3389/frtra.2023.1182534)
Supplement: Supplementary file 1 [file Datasheet1.pdf]

Supplementary Table 1: Pre-transplant MCS and Inotrope Support

	Heart Transplant n (%)
**PRE-TRANSPLANT MCS TYPE**	
ECMO	1 (2)
IABP	26 (54)
IABP+ECMO	2 (4)
IABP+LVAD	1 (2)
IABP+ LVAD+ECMO	1 (2)
LVAD	7 (15)
NA	10 (21)
**PRE-TRANSPLANT INOTROPE**	
DOBUTAMINE	5 (10)
DOBUTAMINE + DOPAMINE	11 (23)
DOPAMINE	1 (2)
MILRINONE	12 (25)
MILRINONE + DOBUTAMINE	3 (6)
MILRINONE + DOPAMINE	1 (2)
MILRINONE + OTHER	1 (2)
NONE	14 (29)

MCS – mechanical circulatory support, ECMO – extracorporeal membrane oxygenation; IABP – intraaortic balloon pump; LVAD – left ventricular assist device

Supplemental Table 2: Antibiotic exposure up to 3 months pre-transplant*

Class	***Name and Route***	***n (%)***
None		23(47.9)
Glycopeptides	Vancomycin IV	11(22.9)
	Vancomycin PO	1(2.1)
Penicillins	Ampicillin-Sulbactam IV	2(4.2)
	Amoxicillin PO	2(4.2)
	Piperacillin-Tazobactam IV	2(4.2)
Cephalosporins	Cephalexin PO	1(2.1)
	Cefazolin IV	6(12.5)
	Cefoxitin IV	1(2.1)
	Ceftriaxone IV	1(2.1)
	Cefepime IV	4(8.3)
Aminoglycosides	Gentamicin IV	2(4.2)
Macrolides	Azithromycin PO	1(2.1)
Tetracyclines	Doxycycline PO	1(2.1)
Fluoroquinolones	Ciprofloxacin PO	1(2.1)
Other	Clindamycin PO	1(2.1)
	Sulfamethoxazole-Trimethoprim DS PO	1(2.1)
	Metronidazole IV	4(8.3)

IV – intravenous; PO – by mouth; DS – double strength
* No instances of exposures to the following were observed: meropenem, linezolid, aztreonam, cefpodoxime, ceftazidime

Supplementary Figure 1: Correlation of demographic factors and co-morbidities to alpha-diversity, butyrate, and secondary bile acids.

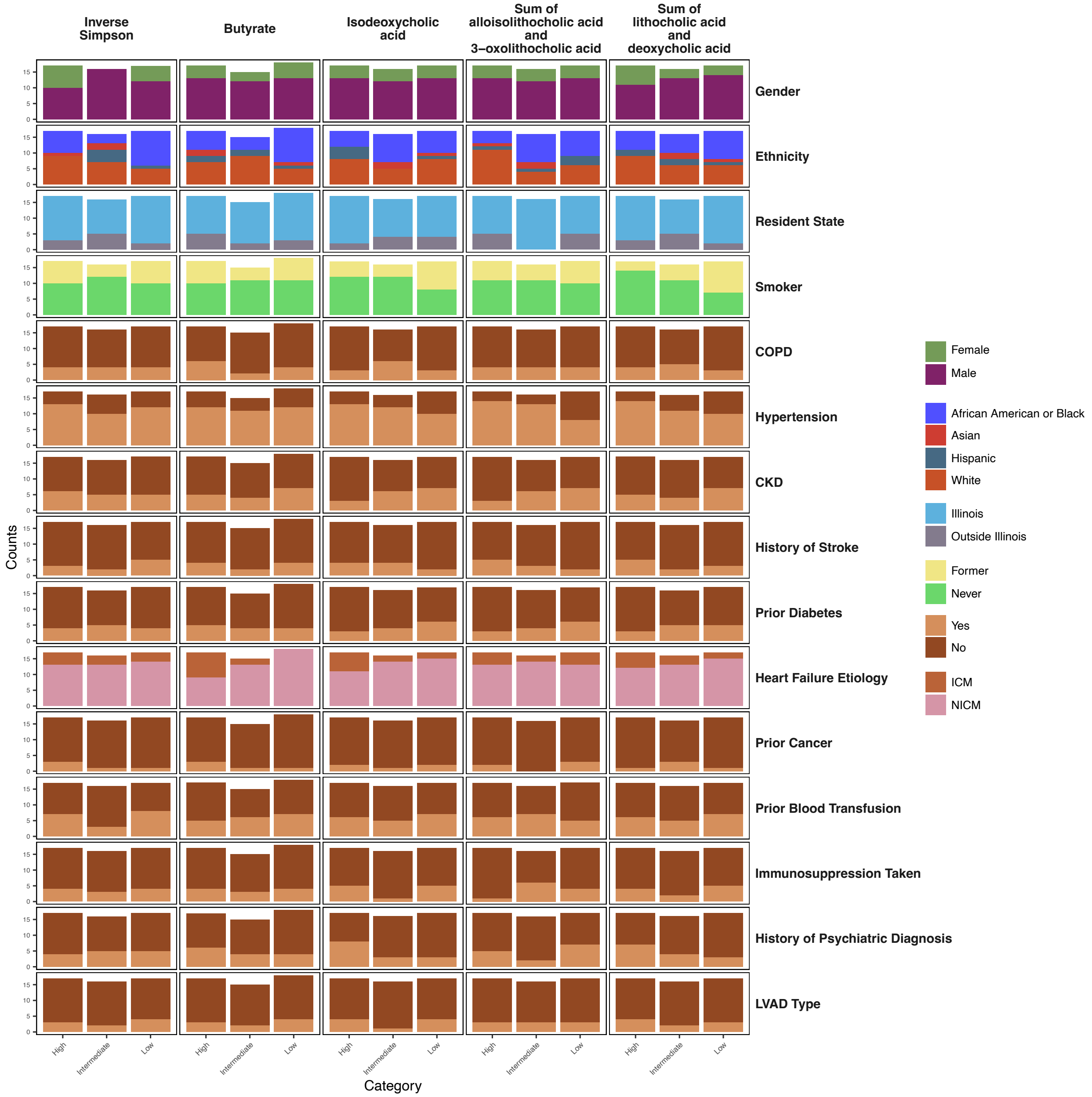

COPD – chronic obstructive pulmonary disease; CKD – chronic kidney disease; LVAD – left ventricular assist device; ICM – ischemic cardiomyopathy; NICM – nonischemic cardiomyopathy

Supplementary Figure 2: Comparison of short and long pre-sample hospitalization alpha **(A)** and beta-diversity **(B)**.

A.

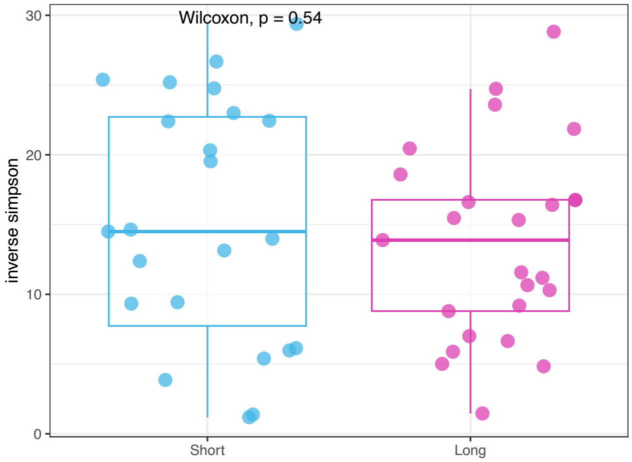

B.

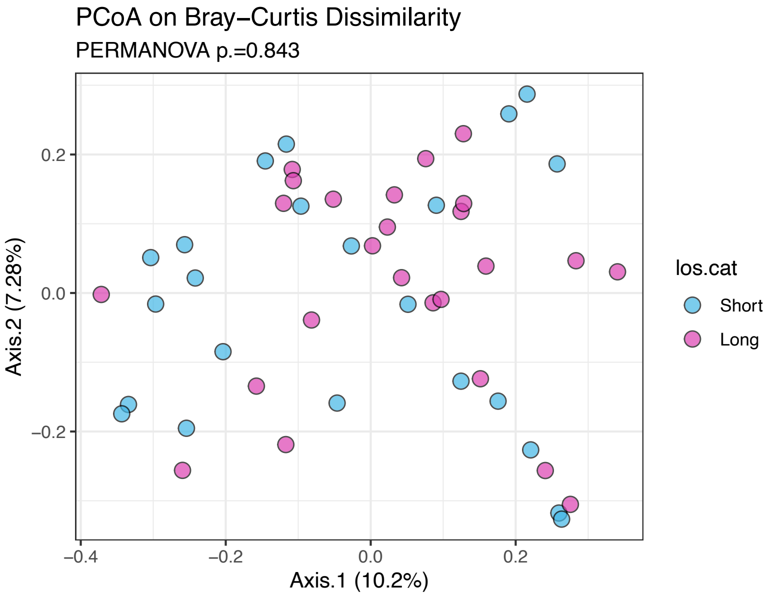

Supplementary Figure 3: Comparison of short chain fatty acids **(A)**, primary bile acids **(B)**, secondary bile acids **(C)**, and derived secondary bile acids **(D)** by pre-sample hospitalization length-of-stay.

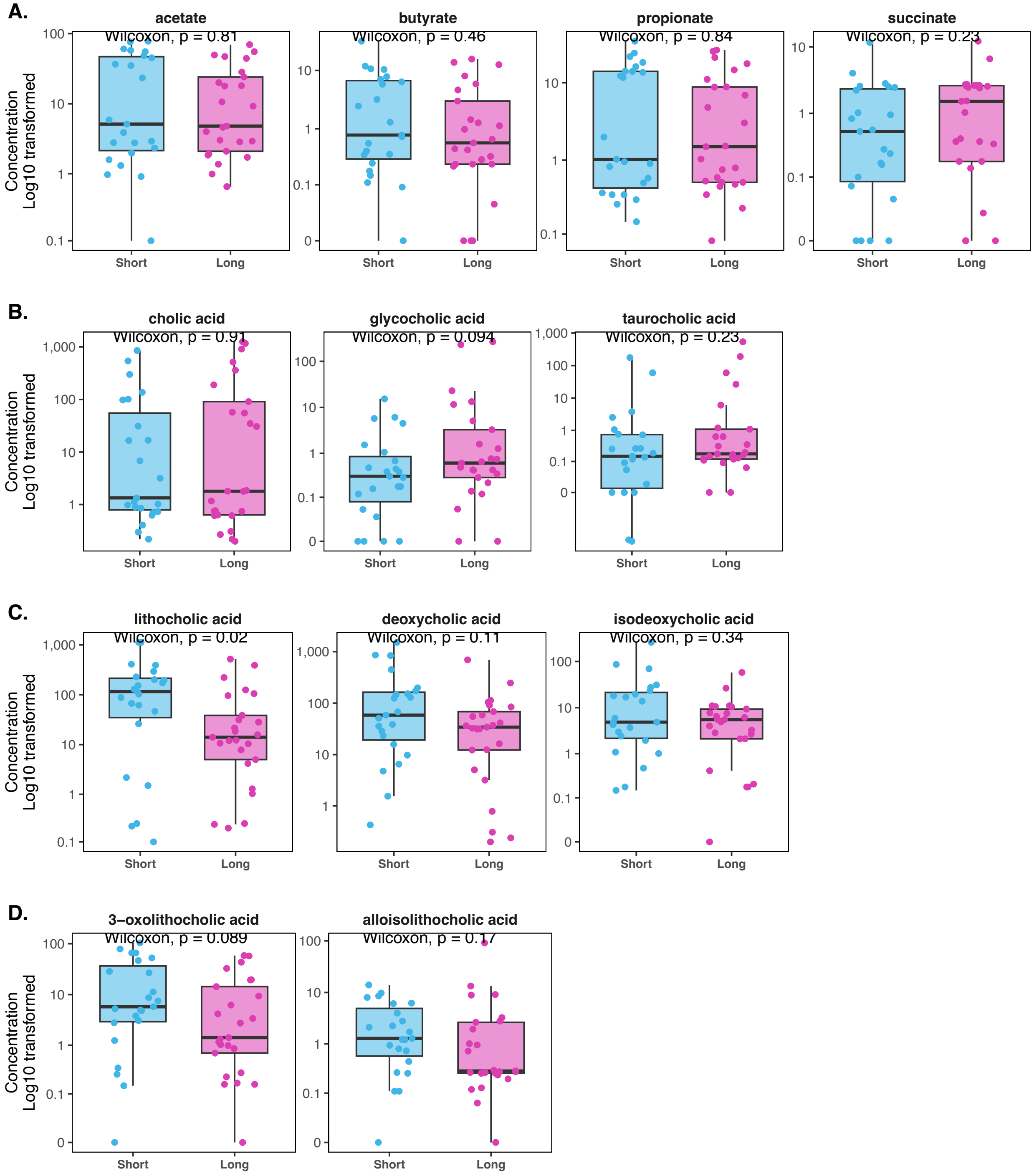

Supplementary Figure 4: Comparison of alpha (A) and beta (B) diversity based on exposure to any antibiotic 7 days prior to sample production.

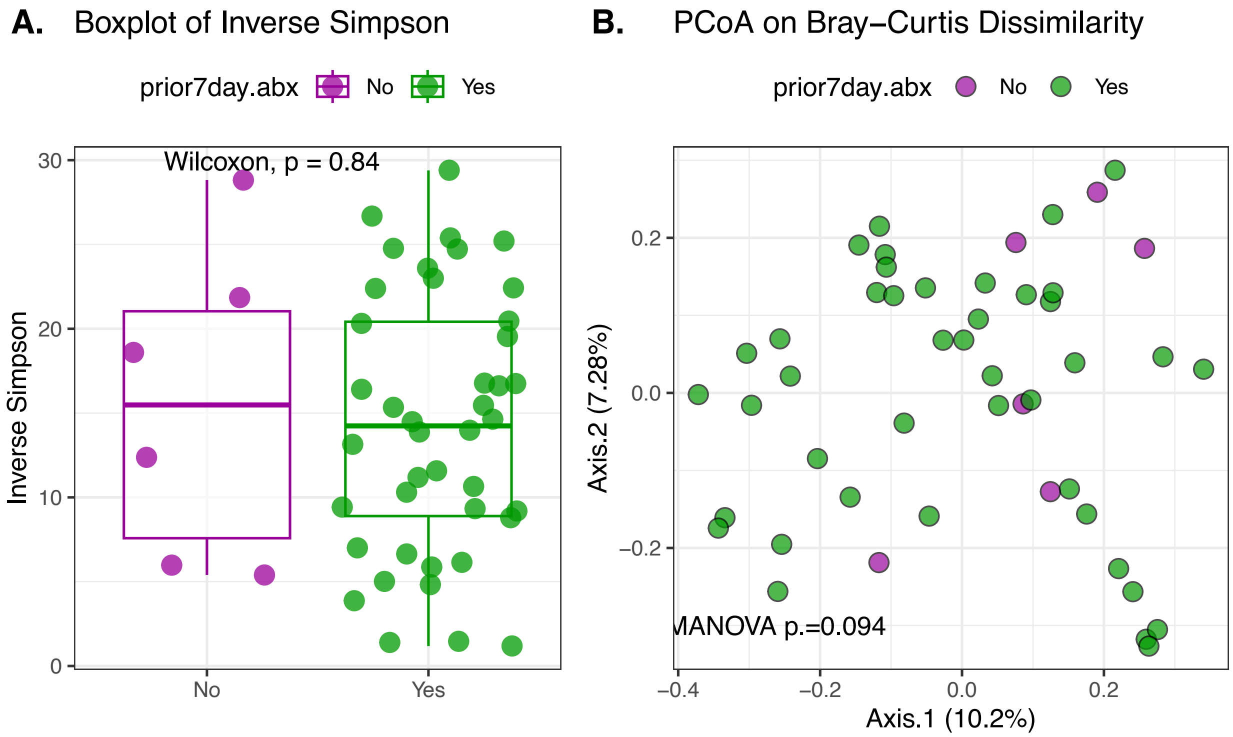

Supplementary Figure 5: Comparison of short chain fatty acids **(A)**, primary bile acids **(B)**, secondary bile acids **(C)**, and derived secondary bile acids **(D)** by exposure to any antibiotic 7 days prior to sample production.

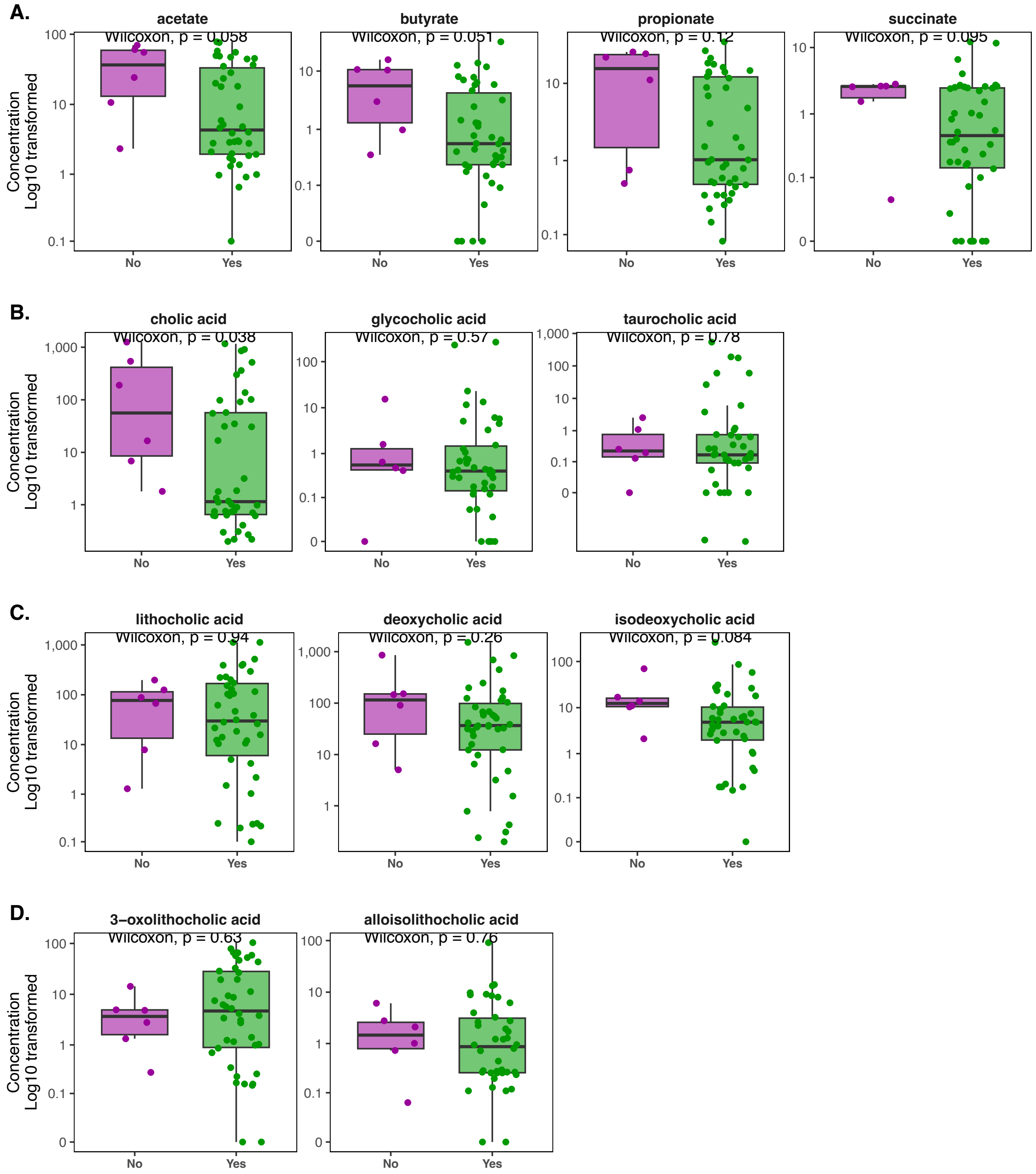

Supplementary Figure 6: Comparison of pre- and post-heart transplant sample alpha-diversity **(A)** and beta-diversity **(B)**.

A.

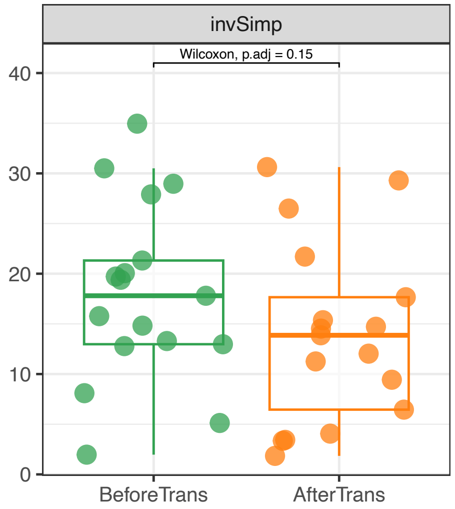

B.

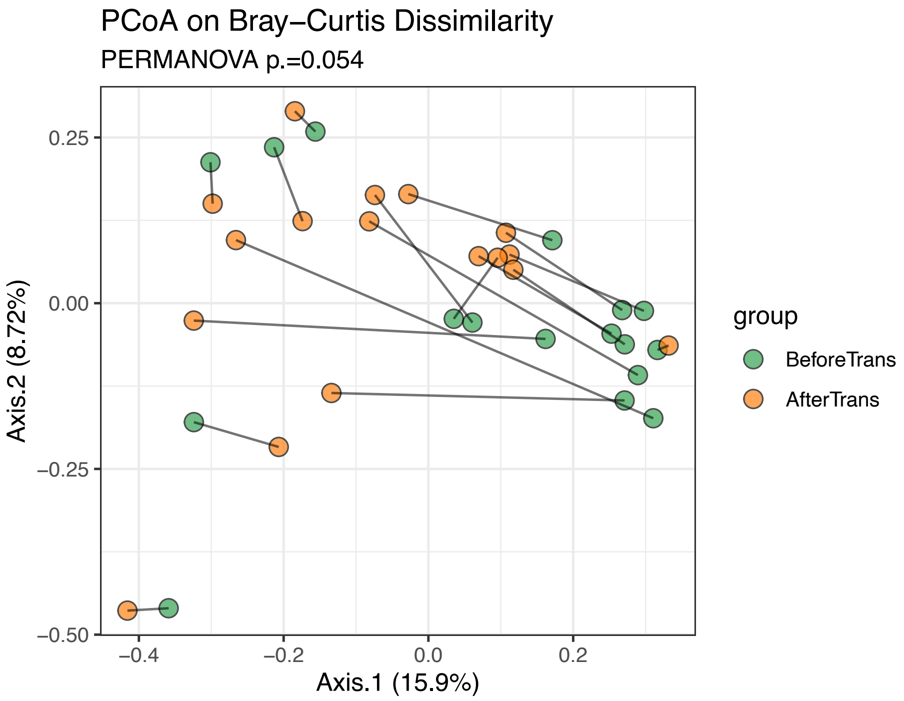

Supplementary Figure 7: Analysis of metabolite production between pre- and post-heart transplant samples showing short chain fatty acids **(A)**, primary bile acids **(B)**, secondary bile acids **(C)**, and modified secondary bile acids **(D)**.

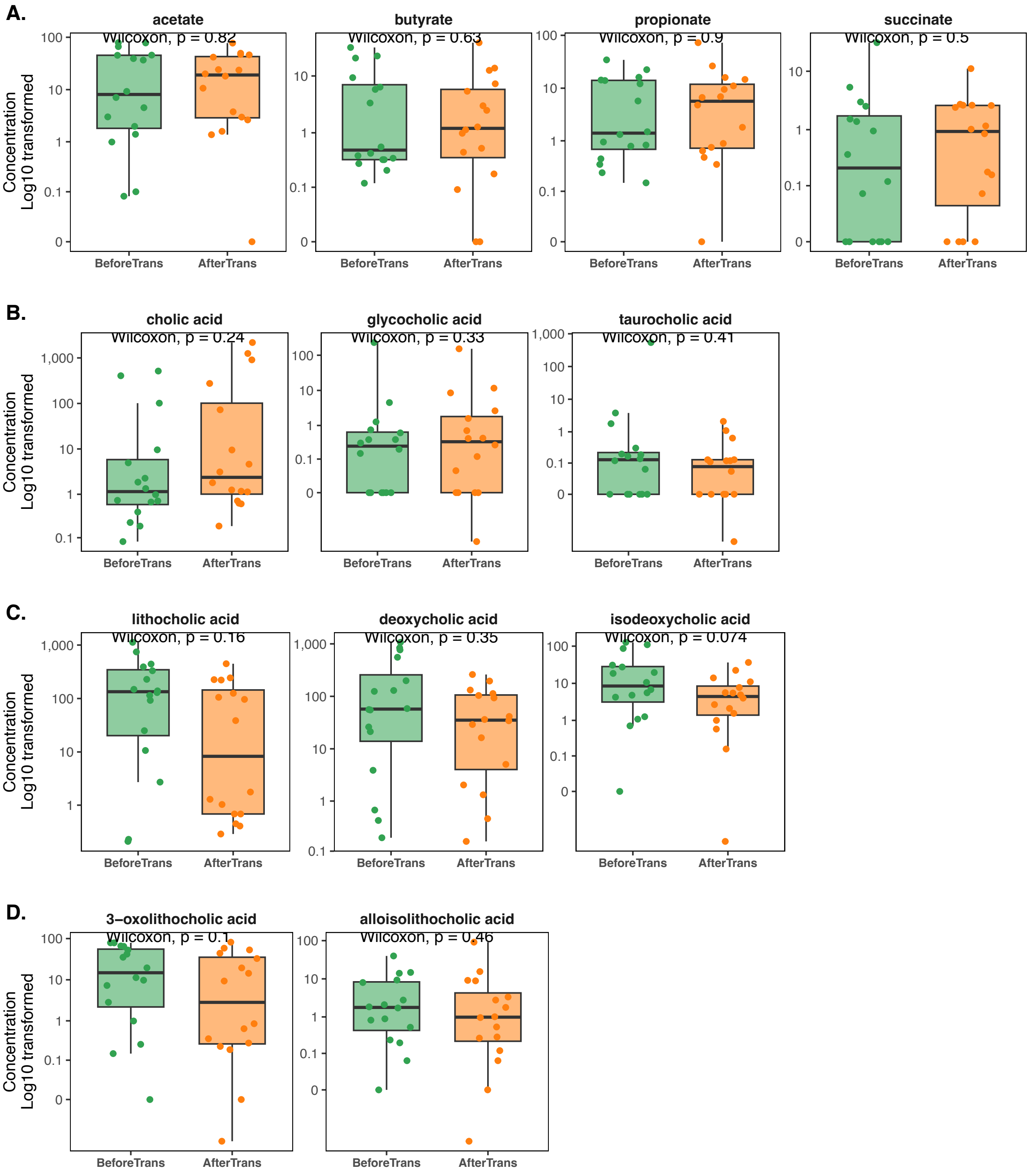

Supplementary Figure 8: Comparison of metabolite production between heart-alone versus multi-organ versus healthy donors showing short chain fatty acids **(A)**, primary bile acids **(B)**, secondary bile acids **(C)**, and modified secondary bile acids **(D)**.

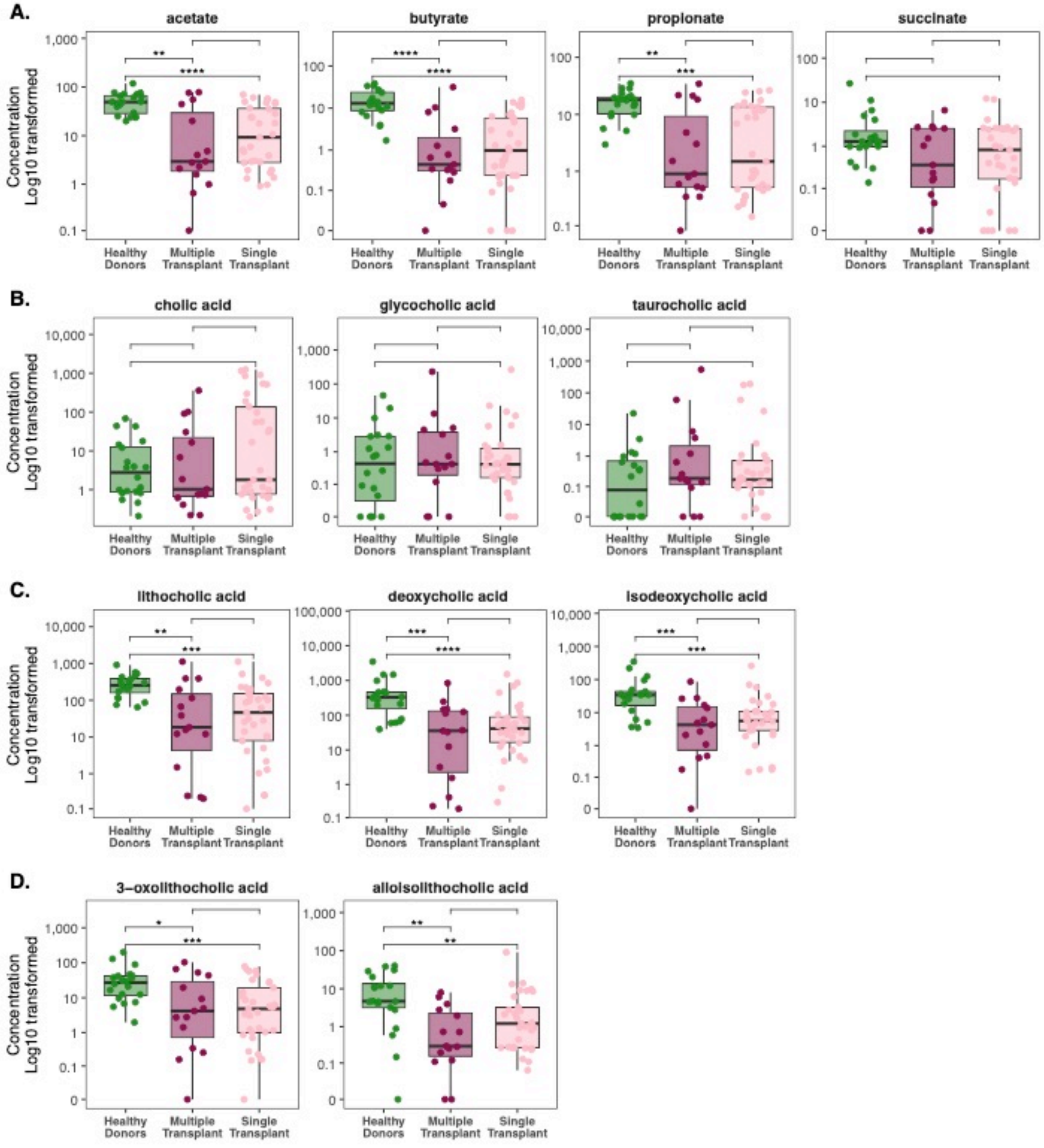

*p<0.05, **p<0.001, ***p<0.0001, ****p<0.00001
